# Potential Application of *p*-Coumaric Acid on Differentiation of C2C12 Skeletal Muscle and 3T3-L1 Preadipocytes—An in Vitro and in Silico Approach

**DOI:** 10.3390/molecules21080997

**Published:** 2016-08-02

**Authors:** Soundharrajan Ilavenil, Da Hye Kim, Srisesharam Srigopalram, Mariadhas Valan Arasu, Kyung Dong Lee, Jeong Chae Lee, Jong Suk Lee, Senthil Renganathan, Ki Choon Choi

**Affiliations:** 1 Grassland and Forage Division, National Institute of Animal Science, Rural Development Administration, Cheonan 330-801, Korea; arulvenil@rediffmail.com (S.I.); srigopalram@rediffmail.com (S.S.); 2 Laboratory of Animal Physiology, Graduate School of Agricultural Science, Tohoku University, Aoba, Sendai 980-8577, Japan; pioioiq@nate.com; 3 Department of Botany and Microbiology, Addiriyah Chair for Environmental Studies, College of Science, King Saud University, Riyadh 11451, Saudi Arabia; mvalanarasu@gmail.com; 4 Department of Oriental Medicine Materials, Dongsin University, Naju 520-714, Korea; leek-d@hanmail.net; 5 Research Center of Bioactive Materials, Institute of Molecular Biology and Genetics, Chonbuk National University, Jeonju 561-756, Korea; leejc88@jbnu.ac.kr; 6 Biocenter, Gyeonggi Institute of Science and Technology, Suwon 443-270, Korea; jslee@gstep.re.kr; 7 Department of Bioinformatics, Marudupandiyar College, Tamilnadu 613-403, India; rsenthil@mpi.edu.in

**Keywords:** coumaric acid, C2C12 and 3T3-L1cells, AMPK-α, PPAR-γ2, PCR and Western blot, computational biology

## Abstract

Coumaric acid (CA) is a phenolic acid of the hydroxycinnamic acid family, and it has many biological functions such as anti-oxidant, anti-inflammatory, antidiabetic, anti-ulcer, anti-platelet, anti-cancer activities, etc. In the present study, we planned to analyse the potential molecular function of CA on skeletal muscle and preadipocytes differentiation using PCR and Western blot techniques. First, we analysed the impact of CA on C2C12 skeletal muscle differentiation. It revealed that CA treatment inhibited horse serum-induced skeletal muscle differentiation as evidenced by the decreased expression of early myogenic differentiation markers such as Myogenin and myoD via the AMP activated protein kinase- alpha AMPK-α mediated pathway. Furthermore, the level of lipid accumulation and changes in genes and protein expressions that are associated with lipogenesis and lipolysis were analyzed in 3T3-L1 cells. The Oil Red O staining evidenced that CA treatment inhibited lipid accumulation at the concentration of 0.1 and 0.2 mM. Furthermore, coumaric acid treatment decreased the expression of main transcriptional factors such as CCAAT/enhancer binding protein-alpha (C/EBP-α) and peroxisome proliferator-activated receptor gamma-2 (PPAR-γ2). Subsequently, CA treatment decreased the expression of sterol regulatory element binding protein-1 (SREBP-1), fatty acid synthase (FAS), acetyl CoA carboxylase (ACC) and adiponectin. Finally, we identified conformational changes induced by CA in PPAR-γ2 using computational biology tools. It revealed that CA might downregulate the PPAR-γ2 expression by directly binding with amino acids of PPAR-γ2 by hydrogen at 3.26 distance and hydrophobic interactions at 3.90 contact distances. These data indicated that CA suppressed skeletal muscle and preadipocytes differentiation through downregulation of the main transcriptional factors and their downstream targets.

## 1. Introduction

Skeletal muscle cells constitute around 40% of individual body mass and play a vital role in locomotion and whole body metabolism. Skeletal muscle cells are the major site of human energy production, and they are responsible for approximately 70% of glucose utilization and lipid homoeostasis [1,2] Skeletal muscle development is a strictly regulated process involving the specification of mesodermal into myoblasts, following the differentiation and fusion, finally changed into multinucleated myotubes. Myogenic regulatory factors (MRFs) play a main role in muscle fusion during differentiation [3,4]. Among these factors, basic helix-loop-helix (bHLH) transcription factors, Myogenin, myogenic differentiation-1 (MyoD), myogenic factors-5 (Myf5) and myogenic regulatory factor-4 play a critical role in muscle formation. MyoD and Myf5 are needed for the development of skeletal muscle and play unique roles in the development of epaxial and hypaxial muscle, respectively [5,6]. These factors activate the differentiation of myogenic progenitors into myoblast. Furthermore, it differentiates into myotubes through Myogenin. In addition, MyoD stimulates myoblast differentiation by regulating the cell cycles [7].

Adipocytes are the major cellular component of fatty tissue. Excess of fat is stored in adipocytes as triglycerides and it increased triglyceride levels in plasma and tissues like liver and muscles that make pathological dysfunctions of these tissues [8,9]. Fat accumulation and adipocyte differentiation are associated with the development of obesity [10]. In addition, it is involved in the maintenance of energy balance and lipid homoeostasis by releasing free fatty acids and storing triacylglycerols in response to changes in energy demands. Increases in adipocyte numbers (hyperplasia) and sizes (hypertrophy) are closely associated with obesity. Hypertrophy of adipocyte is considering as the main factor in adult obesity. However, hyperplasia of adipocytes in obese adults sometimes occur secondary to adipocyte hypertrophy because adipocytes have the capacity to secrete paracrine growth factors that can stimulate adipocyte hyperplasia [11] CCAAT/enhancer binding protein-β (C/EBP-β), CCAAT/enhancer binding protein-alpha (C/EBP-α), nuclear receptor peroxisome proliferator-activated receptor gamma-2 (PPAR-γ2) are main transcriptional factors that regulate adipogenesis. Subsequently, it activates adipocyte specific genes such as adipocyte binding protein (aP2), lipoprotein lipase (LPL), leptin, adiponectin and fatty acid synthase (FAS), which lead to stimulating lipid accumulation within the cells [12,13,14].

*p*-Coumaric acid is a phenolic acid of the hydroxycinnamic acid family. It biologically synthesised by the shikimate pathway with phenylalanine and tyrosine as precursors [15]. Coumaric acid (CA) is found commonly in plants and mushrooms in free or bound form. Both states of CA are widely distributed in fruits, vegetables, cereals and ryegrasses. Coumaric acid modulates glucose uptake and lipid metabolism through the AMP activated protein kinase (AMPK) signalling pathway in L6 skeletal muscle [16]. ortho coumaric acid (*o*-CA) inhibited adipocyte differentiation and promoted adiponectin secretion [17]. CA has various biological functions such as anti-oxidant, anti-inflammatory, antidiabetic mellitus, anti-ulcer, anti-platelet, anti-cancer activities, and antimicrobial properties. Furthermore, it prevents atherosclerosis, oxidative cardiac damage, UV-induced damage to ocular tissues, neural injury and gout [18,19,20,21,22,23,24,25]. The present study is planned to investigate the effect of CA on C2C12 and 3T3-L1 cells differentiation.

## 2. Results

### 2.1. Cytotoxicity of CA on C2C12 and 3T3-L1 Cells

**Figure 1:**
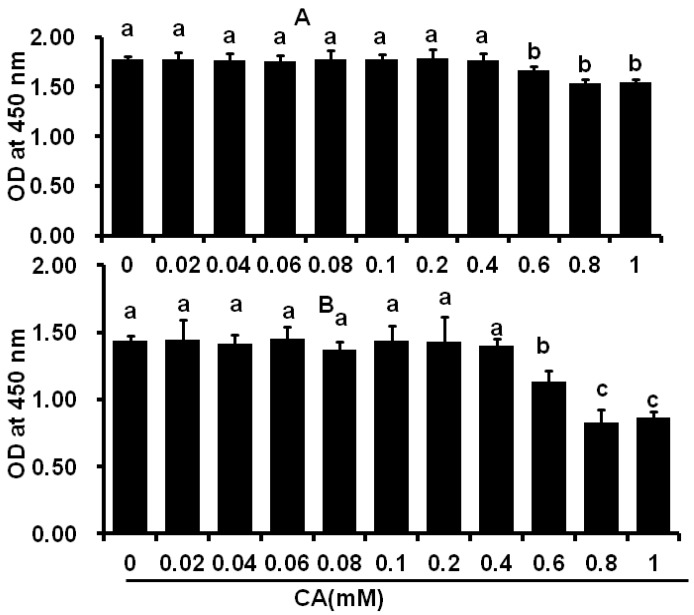
Cytotoxicity of coumaric acid on C2C12 skeletal muscle and 3T3-L1 preadipocytes. C2C12 skeletal and 3T3-L1 cells were incubated with coumaric acid at 0.02, 0.04, 0.06, 0.08, 0.1, 0.2, 0.4, 0.6, and 1 mM concentrations for 48 h. Coumaric acid (CA) did not possess any cytotoxicity effect on skeletal muscles and preadipocytess between the concentration ranges from 0.02 mM to 0.4 mM. (**A**) coumaric acid on C2C12 skeletal muscle; and (**B**) coumaric acid on 3T3-L1 preadipocytes. The results represent the mean ± SEM of six replicates. The different letters—a, b, c—within a treatment indicates significant differences (*p* < 0.05).

The C2C12 and 3T3-L1 cells were treated with different concentrations of CA for 48 h and exhibited no cytotoxic effects up to the concentration of 0.4 mM (Figure 1A,B). However, higher CA levels increased cytotoxicity and decreased cell viabilities.

### 2.2. Coumaric Acid Inhibits Myotube Formation and Early Differentiation Markers in C2C12 Cell

**Figure 2:**
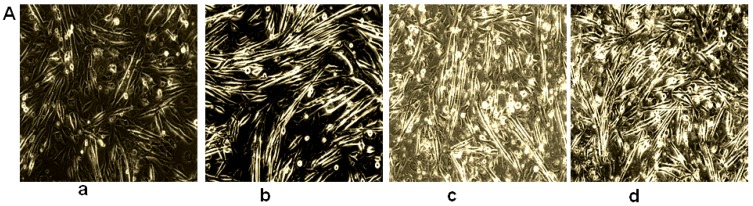

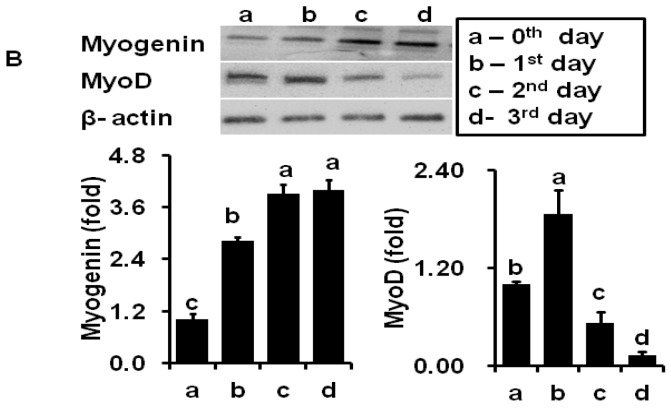
Impact of horse serum on myoblast formation and myogenic markers. (**A**) microscopic views of myoblast differentiation in experimental cells; and (**B**) Western blot analysis of Myogenin and MyoD (myogenic differentiation-1) expression in 2% horse serum (HS) media (a—day 0; b—1st day; c—2nd day; d—3rd day). The results represent the mean ± SEM of three replicates. The different letters—a, b, c, d,—within a treatment indicates significant differences (*p* < 0.05).

**Figure 3:**
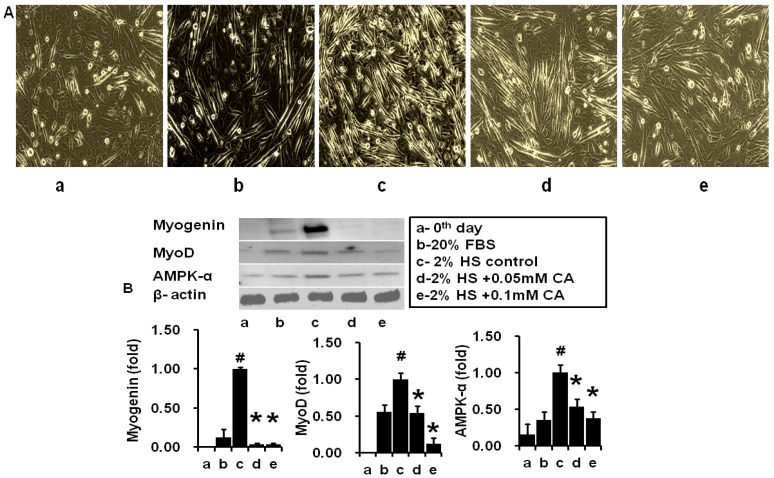
Impact of coumaric acid on myoblast formation and myogenic markers. (**A**) microscopic views of myoblast differentiation in experimental cells; (**B**) Western blot analysis of Myogenin, MyoD and AMPK-α (AMP activated protein kinase-alpha) expression in experimental cells (a—day 0; b—20% FBS; c—2% HS (Horse serum; d—2% HS + 0.05 mM CA; e—2% HS + 0.1 mM CA). The results represent the mean ± SEM of three replicates. ^#^ *p* < 0.05 level significance as compared to day 0 and 20% FBS (Bovine fetal serum) supplemented cells; * *p* < 0.05 level significance as compared to control cells.

Figure 2 and Figure 3 showed HS and CA impact on morphological changes in C2C12 cells. Here, we noted that myoblast differentiation was decreased with increased concentrations of CA. However, significant inhibition was observed at the concentration of 0.05 and 0.1 mM. The cell shape was gradually changed into the new elongated structure by the horse serum treatment in a time dependent manner (Figure 2A). However, CA treatment decreased morphological changes and almost maintained the native shape of the C2C12 cells in a concentration dependent manner (Figure 3A). Furthermore, we determined early differentiation markers such as Myogenin and myoD expression in horse-serum treated cells. It revealed that Myogenin and myoD expressions had been changed in a time dependent manner (0, 1, 2, and 3 days). Myogenin level was prominently expressed on day 2, whereas MyoD level was prominently expressed on day 1 (Figure 2B). However, CA treatment (0.05 and 0.1 mM) significantly reduced both Myogenin and myoD levels on day 2, as compared with control cells. In addition, AMPK-α expression was downregulated by CA treatment at the concentration of 0.05 and 0.1 mM as compared with control cells (Figure 3B). These data suggest that CA inhibited C2C12 cells differentiation through the AMPK-α mediated pathway.

### 2.3. Coumaric Acid Inhibits Differentiation and Lipid Accumulation in 3T3-L1 Cells

**Figure 4:**
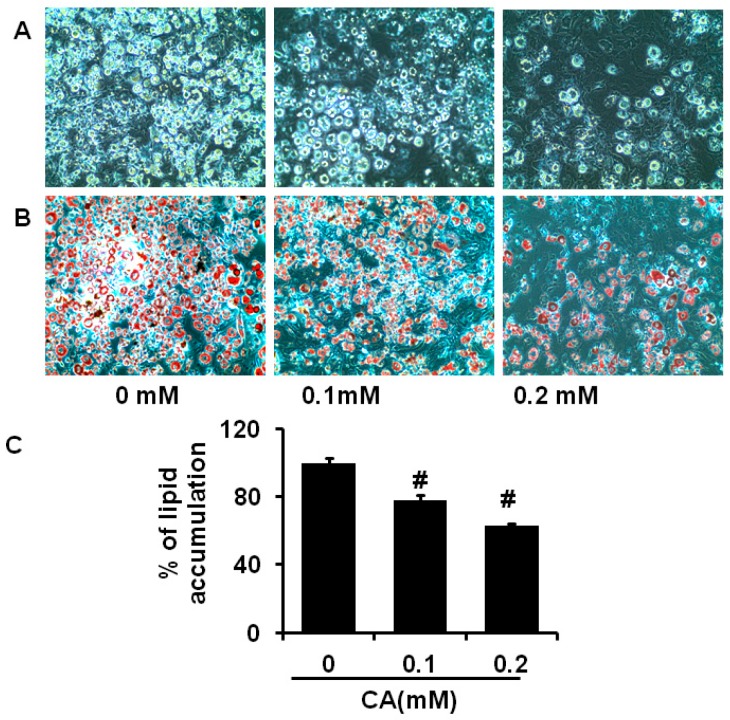
Coumaric acid inhibits differentiation and lipid accumulation in 3T3-L1 preadipocytes. Coumaric acid treatment decreased lipid droplet accumulation in a dose dependent manner. (**A**) microscopic views of experimental adipocytes; (**B**) Oil red O stained lipid droplets in adipocytes; and (**C**) % of lipid accumulation in experimental adipocytes. The results represent the mean ± SEM of three replicates. ^#^ *p* < 0.05 level significance as compared to control cells (0 concentration).

Furthermore, we analysed whether CA could activate or inhibit adipocyte differentiation and lipid accumulation in 3T3-L1 cells by Oil red O staining method. The adipocyte differentiation is directly associated with Oil red O stained cells due to accumulation of cytoplasmic lipid droplets. Microscopic views of adipocytes showed gradual decreases in the lipid droplet accumulation as the concentration of CA increased when compared to control cells (Figure 4A,B). In addition, Oil red O stain was extracted from experimental adipocytes with 100% isopropyl alcohol and measured absorbance intensity. It revealed that CA treatment significantly (*p* < 0.05) decreased lipid accumulation compared to control adipocyte (Figure 4C). These results suggest that CA treatment effectively inhibited lipid accumulation and differentiation in 3T3-L1 cells at 0.1 mM and 0.2 mM.

### 2.4. CA Downregulates Adipogenic and Lipogenic Gene Expression

**Figure 5:**
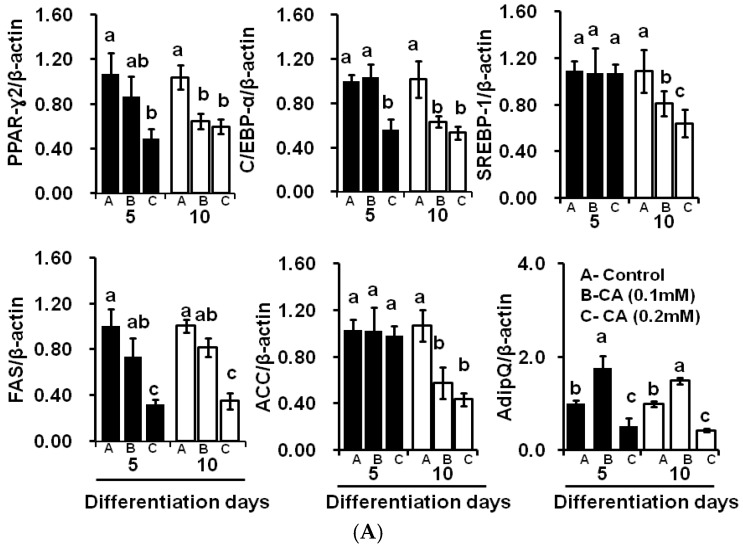

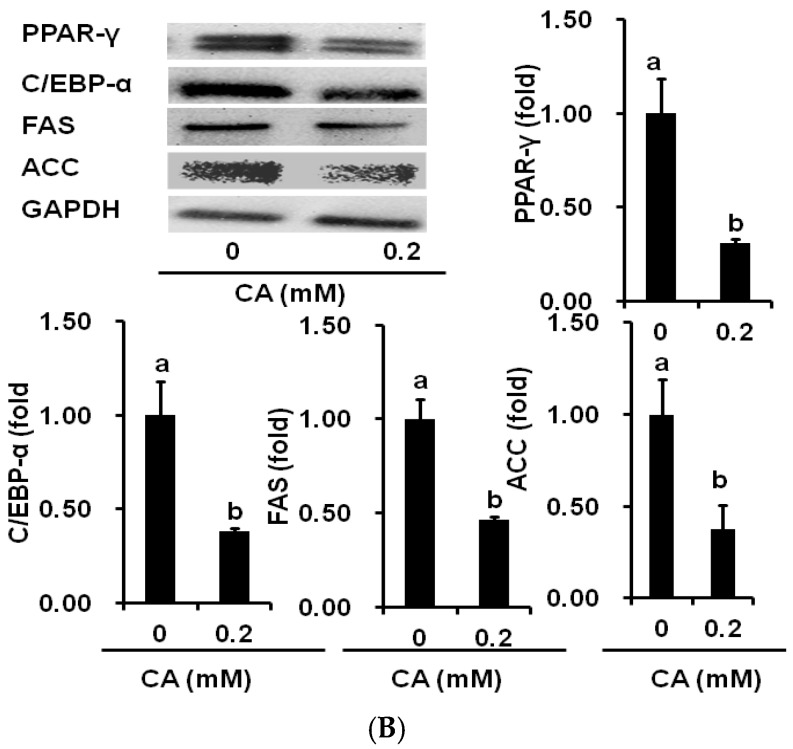
Effects of coumaric acid on peroxisome proliferator-activated receptor gamma-2 (PPAR-γ2), CCAAT/enhancer binding protein-alpha (C/EBP-α), sterol regulatory element binding protein-1 (SREBP-1), fatty acid synthase (FAS), acetyl CoA carboxylase (ACC). CA treatment downregulated the adipogenic and lipogenic mRNA expressions at differentiation periods. (**A**) quantification of mRNA expressions in experimental adipocytes using the qPCR technique. The results represent the mean ± SEM of six replicates. The different letters—a, b, c—within a treatment indicate significant differences (*p* < 0.05); and (**B**) qualitative and quantitative analysis of protein expressions in experimental cells by Western blot and ImageJ software (1.49 version, 32 bit, Wayne Rasband, National Institute of Health, Bethesda, MD, USA). The results represent the mean ± SEM of three replicates. The different letters—a, b—within a treatment indicate significant differences (*p* < 0.05).

Furthermore, we analysed adipogenic genes expressions of control and experimental cells using RT-PCR. These results demonstrated the adipogenic key transcriptional factors like C/EBP-α, and PPAR-γ2 was decreased in cells by CA treatment as compared to those in the control cells. In addition, CA treatment downregulated adiponectin, ACC, and FAS mRNA expression as compared to respective day of control adipocytes (Figure 5A). Furthermore, we investigated protein expression patterns in the experimental cells by Western blot technique. Results suggested that CA treatment decreased key transcriptional factors such as PPAR-γ2, C/EBP-α, and lipogenic enzymes such as FAS and ACC expression on the tenth day of differentiation (Figure 5B). These results strongly suggest that CA treatment significantly downregulated key adipogenic and lipogenic protein expression.

### 2.5. Comparative Study between CA, TRZ, and T0070907 on PPAR-γ2 Expression

**Figure 6:**
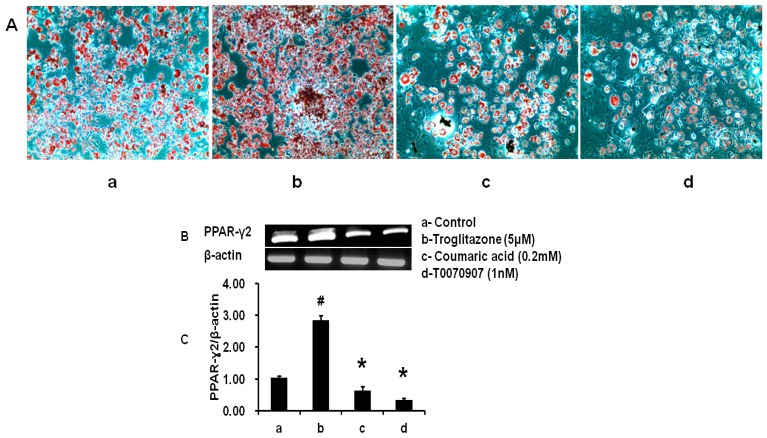
Comparative effects between coumaric acid, troglitazone (TRZ) and T0070907 on lipid accumulation and PPAR-γ2 mRNA expression. (**A**) lipid droplets stained cells by Oil red O staining method; (**B**) qualitative analysis of PPAR-γ2 mRNA expression; and (**C**) quantitative analysis of PPAR-γ2 mRNA expression (a—control; b—troglitazone (5 μM); c—coumaric acid (0.2 mM); d-T0070907 (1 nM). The results represent the mean ± SEM of six replicates. ^#^ *p* < 0.05 compared to control adipocytes; * *p* < 0.05 compared to control and TRZ treatment.

The impacts of CA, troglitazone (TRZ-agonist), and T0070907 (antagonist) on lipid accumulation and PPAR-γ2 expression were analysed. Troglitazone treatment increased lipid accumulation and PPAR-γ2 expression compared to control cells. However, lipid accumulation and PPAR-γ2 expression were decreased in cells treated with 1 nM of T0070907. Similarly, CA treatment significantly reduced lipid accumulation and PPAR-γ2 expression as compared to control or TRZ treatment (*p* < 0.05). These data conclude that CA could effectively inhibit adipocyte differentiation via downregulating PPAR-γ2 (Figure 6).

### 2.6. Molecular Modelling for CA with PPAR-γ2

**Figure 7:**
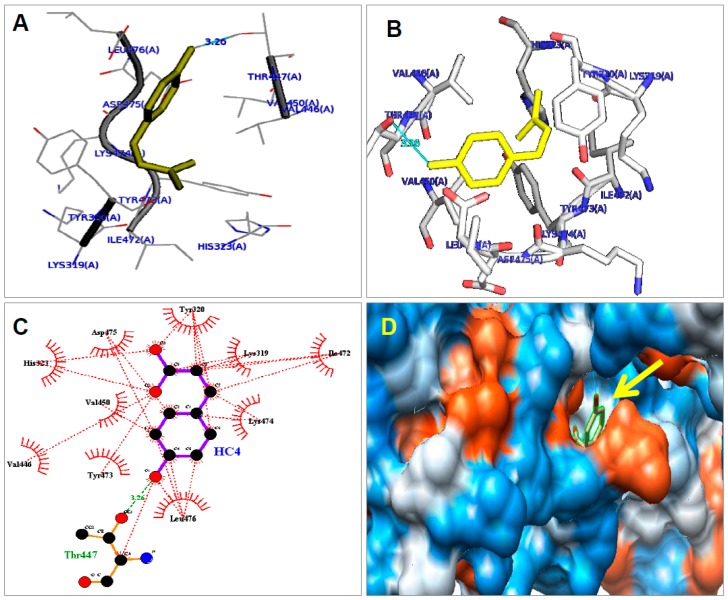
Interaction of coumaric acid (FA) with amino acids in PPAR-γ2 receptor. (**A**,**B**) coumaric acid with PPAR-γ2 in stick model; (**C**) schematic diagram of PPAR-γ2 with coumaric acid; and (**D**) electrostatic surface of PPAR-γ2 alongside of the LXXLL (L-leucine, X- any amino acids) motif with CA.

Identification and screening of bioactive molecules and their binding sites in the receptor are challenging for drug development. Therefore, we planned to find out the binding ability of CA with PPAR-γ2 receptor using computational biology tools. Figure 7A,B represents the amino acids of PPAR-γ2 (ILE472-476) helix in the monomer (PDB id: 1prgA) were exhibited in stick model. The structure of CA (HC4) and the residues in the PPAR-γ2 ligand binding domain were represented (with affinity score –5.5 and Root mean square deviation (RMSD) 5.038). Figure 7C indicates a schematic diagram of PPAR-γ2 with CA. Coumaric acid interacted with different amino acids of PPAR-γ2 by hydrogen and hydrophobic bonding. Hydrogen bond was indicated by dashed lines in green between atoms involved (Thr447 with 3.26 distance) and rest of the interactions were hydrophobic within 3.90 maximum contact distance (residues Lys 319, Tyr 320, His 321, Val 446&450, Lys 474, Asp 475 and Leu 476). Electrostatic surface of PPAR-γ2 monomer along the side of the LXXLL motif with CA is red and blue (yellow arrow) (Figure 7D).

## 3. Discussion

### 3.1. Coumaric Acid on C2C12 Cell Differentiation

Skeletal muscle plays critical functions in the muscle movement, supporting respiration and maintaining homoeostasis. The loss of muscle cells mass or its functions is associated with ageing and a variety of diseases [26]. Myogenic regulatory factors (MRFs) such as Myogenin, MyoD, MRF4 Myf5, and basic-helix-loop-helix transcriptional factors regulate the myogenesis [27,28,29,30]. Among these, MyoD determine the myogenic lineage of muscle progenitor cells. However, Myogenin can drive the terminal differentiation and fusion of myoblast into myotubes, finally developing the myofibers. MyoD is positively regulated by numerous biochemical changes and interactions like ubiquitination, acetylation and phosphorylation. In addition, MyoD is negatively regulated by methylation [31,32,33,34,35,36,37]. Overexpression of MyoD is able to stimulate skeletal cell differentiation [38]. MyoD is prominently expressed in myotubes and joins with Myogenin to regulate the expression of genes that are responsible for differentiation [39]. In this study, the C2C12 cells treated with different concentration of CA inhibited horse serum induced morphological changes, and it indicates that CA effectively inhibits myoblast differentiation as evidenced by the decreased expression of early myogenic differentiation markers such as Myogenin and MyoD.

AMP-activated protein kinase (AMPK) is a heterotrimeric enzyme that composed of α, β and γ subunits. It plays the main role in the regulation of energy metabolism [40,41]. AMPK has recognized its regulatory roles in gene expression, cell differentiation and tissue development [42,43]. The role of AMPK in muscle has been well established already, and it promotes muscle protein degradation, autophagy and inhibition of protein synthesis [44]. Expression of AMPK protein can promote Myogenin expression and myogenesis, whereas AMPK inhibition downregulates Myogenin transcription and myogenesis via phosphorylation of HDAC5 mediated mechanism especially AMPK-α1 [45,46]. Here, we noted that CA treatment decreased AMPK-α protein expression as compared with control cells. As we discussed early, AMPK activation could promote the myotube formation and myogenesis. In our study, we noted that CA treatment inhibited myoblast differentiation as compared with control cells. It indicates that the CA could inhibit myoblast differentiation through downregulation of AMPK-α mediated pathway.

### 3.2. Coumaric Acid on 3T3-L1 Cell Differentiation

CA treatment was significantly reduced differentiation as well as lipid accumulation in 3T3-L1 cells as compared with control cells. Furthermore, we noted that the CA treatment effectively inhibited PPAR-γ2 and C/EBP-α mRNA along with its protein expression. Increased fat cell numbers as well as fat cell size are closely associated with obesity [47]. The number of adipocytes present in an organism is determined to a large degree by the adipocyte differentiation process that produces mature adipocytes from preadipocytes. The PPAR-γ and C/EBP-α are the most important crucial factors in adipogenesis and lipogenesis [48,49]. Ectopic expression of PPAR-γ is enough to induce C/EBP-α and fulfill the differentiated adipocytes development from preadipocytes. However, the initial induction of C/EBP-α is based on PPAR-γ expression. Furthermore, C/EBP-α, in turn, is recognized to strengthen the PPAR-γ expression. Many specific genes are needed for adipocyte differentiation and lipid accumulation, among them; PPAR-γ and C/EBP-α are considered very important for the maintenance of adipocytes in the differentiated states [50,51]. For additional evidence, we performed molecular docking for CA with PPAR-γ2, and it shows how CA inhibits PPAR-γ2 expression. We observed that CA interacted with amino acids of PPAR-γ2 by hydrogen and hydrophobic interactions. This result suggests that CA downregulated PPAR-γ2 expression through hydrogen and hydrophobic interactions with the PPAR-γ2 receptor domain. These results are consistent with our experimental results. CA treatment decreased expression of PPAR-γ2 and lipid accumulation, whereas TRZ treatment increased expression of PPAR-γ2 and lipid accumulation.

The SREBP-1 is a transcriptional factor that is involved in lipid metabolism [52], and it regulates FAS and is an additional regulator of adipogenesis. SREBP-1 stimulates preadipocytes differentiation and their genes associated with fatty acid metabolism [53]. In the current study, CA treatment decreased SREBP-1 mRNA expression as compared with control cells, which suggested that CA treatment inhibited adipocyte differentiation and lipid accumulation through altering the transcriptional factors involved at different stages of differentiation. PPAR-γ2, C/EBP-α and SREBP-1 drive adipocyte-specific genes in coordination such as FAS, ACC and FABP4, which determine the later stage of adipocyte differentiation and biosynthesis of fatty acids and triacylglycerols [54]. FAS and ACC are primary enzymes that are regulating fatty acid synthesis [55,56]. The findings of the present study revealed that FAS and ACC expression were inhibited by CA treatment. This suggested that CA inhibits preadipocytes differentiation and lipid accumulation through inhibiting the expression or activity of adipogenic transcriptional factors and their related genes.

Adiponectin is an important hormone secreted in fully differentiated adipocytes. Adiponectin modulates several metabolic processes such as adipocyte differentiation, fatty acid oxidation and insulin responsive transport, and its expression is negatively correlated with adipogenesis [57]. Thus, our data suggest that CA treatment effectively inhibited differentiation and lipid accumulation process through attenuating the adiponectin level.

## 4. Materials and Methods

### 4.1. Cell Culture and Chemicals

The C2C12 and 3T3-L1 cell line were obtained from the American Type Culture Collection (Rockwille, MD, USA). For cell culture, Dulbecco modified Eagle medium (DMEM) and fetal bovine serum (FBS) were procured from Gibco-BRL (Gaithersburg, MD, USA). Kits for mRNA extraction and RT-PCR kit were purchased from Invitrogen (Carlsbad, CA, USA).

### 4.2. Cytotoxicity Assay

Water soluble tetrazolium (WST; 2[2-Methoxy-4-nitrophenyl]-3[4-Nitrophenyl]-5-[2,4-disulfophenyl]-2-*H*-tetrazolium monosodium salt) was used in cytotoxicity assays. Cells were seeded into 96-well culture plates at a density of 1 × 10^4^ cells/well in growth media. After 24 h of culturing, cells were exposed to different concentrations of CA and incubated at 37 °C with 5% CO_2_ for 48 h. The intensity of colour in each well was measured at 450 nm using a spectra count ELISA plate reader (Packard Instrument Co., Downers Grove, IL, USA).

### 4.3. Differentiation Induction Protocol for C2C12 Skeletal Muscle

The cells were seeded in 6-well plates contain 20% FBS in DMEM at a density of 4 × 10^4^ and incubated at 37 °C with 5% CO_2_. After 80%–90% confluency, the growth media was replaced by differentiation medium (2% horse serum). For examining the CA effect on C2C12 skeletal muscle differentiation, the cells were supplied with different concentrations of coumaric acid in differentiation medium until the end of the experimental periods.

### 4.4. Differentiation Induction Protocol for 3T3-L1 preadipocytes

Adipocyte differentiation experiment was carried out according to the method of Choi et al. [58]. To examine the effect of CA on adipocyte differentiation, preadipocytes received different concentrations of CA every 2 days starting from 2 days post confluence until the end of the experiment. The primers used in the study are given in the supplementary file (Table S1).

### 4.5. Quantification of Lipid Content by Oil Red O Staining

3T3-L1 adipocytes were fixed with 10% formalin for 1 h and rinsed with 40% isopropanol. After rinsing, 3 mL of Oil red O staining solution was added to each well and incubated at room temperature for 15 min. After washing thrice with distilled water, adipocytes were photographed under an inverted microscope (CKX41, Olympus Corporation, Tokyo, Japan). Additionally, Oil red O stain was eluted from these adipocytes using isopropanol (100%) and measured at 490 nm [58].

### 4.6. Quantification of Adipogenic Gene Expression Using Quantitative RT-PCR

Total RNA was extracted from experimental samples by MagDEA RNA 100(GC) reagent using automated machine Magtration system 12GC plus (PSS, G180M0002-01, Chiba, Japan). Total RNA has quantified with UVS-99 micro volume UV/Vis spectrometer-ACT gene (ACTGene, Piscataway, NJ, USA). One microgram of RNA was used to synthesis the cDNA by kit protocol (iScript cDNA synthesis, Bio-Rad, Hercules, CA, USA). General PCR was carried out on an AB 9700 PCR system (Applied Biosystems, Foster City, CA, USA). The gene expression patterns were quantified using SYBR Green-based real-time PCR (CFX96 qRT-PCR, Biorad, Seoul, Korea). All expression levels were normalized to the housekeeping gene β-actin [58].

### 4.7. Protein Extraction and Immunoblotting

Protein was extracted from experimental cells with radioimmunoprecipitaion assay buffer (RIPA lysis buffer) with 1X protease inhibitor cocktail (Roche, Basel, Switzerland). Cells were washed with PBS thrice. Then, 300 μL of RIPA lysis buffer/well was added and incubated at 4 °C for 5 min. Then, the cells were scraped rapidly using a cell scraper and collected the cell lysate into clean tubes. The cell lysate was centrifuged at 4 °C for 10 min for removal of undesired precipitates. Protein concentration was quantified by the Bio-Rad protein assay kit (Bio-Rad, Seoul, Korea). Furthermore, equal amounts of proteins were separated by SDS-PAGE (12%) (Mini- protein TGX precast gels, Biorad, Seoul, Korea) and blotted onto polyvinylidene difluoride (PVDF) membranes (iBlot gel transfer stacks, Novex, Life Technology, Waltham, MA, USA). Immunoblotting was performed (primary antibody incubated at 4 °C for overnight) according to Western breeze chemiluminescence protocol (Invitrogen, Seoul, Korea) using rabbit monoclonal antibody against target proteins PPAR-γ2, C/EBP-α, FAS and MyoD (Cell Signaling Technology, Danvers, MA, USA) and Myogenin was from Santa Cruz Biotechnology, Inc. (Dallas, TX, USA). Band signals were detected with an enhanced chemiluminescence kit (Bio-Rad) on a chemiluminescence imaging system (Davinch Chemiluminescence, Seoul, Korea). The intensity of antibody reacted bands was quantified with ImageJ software (1.49 version, 32 bit, Wayne Rasband, National Institute of Health, Bethesda, MD, USA).

### 4.8. Molecular Modeling

Protein and ligand structures were obtained from the protein data bank (PDB) database and Pubchem. Before the interaction study, the Swiss Dock server was used to identify clusters and potential targets [59]. With the cluster details, protein and ligand properties have been prepared by molecular graphics laboratory tools (MGL-1.5.6 version, Scripps Research Institute, Florida, FL, USA). Complex structures were modeled using modeling software’s Pymol (1.1 version, Delano Scientific LLC, San Carlos, CA, USA), Chimera (1.10.1 version UCSF Resources for biocomputing visualization and informatics, NIH, CA, USA), and Ligplot (1.4.5. version, European Bioinformatics Institute, Cambridge CB10 1SD, UK) [60].

### 4.9. Statistical Analysis

Each experiment was carried out in replicates (*n* = 6, *n* = 3). Data were expressed as the mean and standard error of mean (SEM). Statistical analysis was carried out using one-way ANOVAs, multivariate comparisons, and Student’s *t*-tests using the statistical package of social science (SPSS) program (Version 16.0, SPSS, Inc., Chicago, IL, USA). Statistical significance was considered when the *p*-value was less than 0.05.

## 5. Conclusions

We investigated the modulatory effect of CA on C2C12 skeletal muscle cells and 3T3-L1 preadipocytes differentiation. These data suggest that CA treatment inhibited skeletal muscle cell and preadipocytes differentiation through inhibiting the genes that are responsible for myogenesis and adipogenesis. The interaction studies of CA with PPAR-γ2 receptor supported this inhibition of adipocyte differentiation. Taken together, our data have demonstrated that coumaric acid treatment inhibits C2C12 and 3T3-L1 cells differentiation by regulating the transcriptional factors and their downstream targets.

## Supplementary Materials

Supplementary materials can be accessed at: http://www.mdpi.com/1420-3049/21/8/997/s1.
