# Supplementary material for: Potential Application of p-Coumaric Acid on Differentiation of C2C12 Skeletal Muscle and 3T3-L1 Preadipocytes—An in Vitro and in Silico Approach
Source: Molecules. 2016 Aug 2;21(8):997. doi: 10.3390/molecules21080997 (PMC6274435; doi:10.3390/molecules21080997)
Supplement: Supplementary file 1 [file molecules-21-00997-s001.pdf]

Supplementary Materials: Potential Application of *p*-Coumaric Acid on Diffe rentiation of C2C12 Skeletal Muscle and 3T3-L1 Pre-Adipocytes—An in Vitro and in Silico Approach

Soundharrajan Ilavenil, Da Hye Kim, Srisesharam Srigopalram, Mariadhas Valan Arasu, Kyung Dong Lee, Jeong Chae Lee, Jong Suk Lee, Senthil Renganathan and Ki Choon Choi

Table S1. Table list of primers used in the experiment.

Sample No.	Gene Name	Forward Primers	Reverse Primers
1	PPRA-γ2	GTGCTCCAGAAGATGACAGAC	GGTGGGACTTTCCTGCTAA
2	C/EBP-α	GCAGGAGGAAGATACAGGAAG	CAGACTCAAATCCCAACA
3	Adiponectin	CCGTTCTCTTCACCTACGAC	TCCCCATCCCCATACAC
4	SREBP-1	GAAGTGGTGGAGAGACGCTTAC	TATCCTCAAAGGGCTGGACTG
5	FAS	CCCAGCCCATAAGAGTTACA	ATCGGGAAGTCAGCACAA
6	ACC	GGGCTACCTCTAATGGTCTT	CTACCTGATGGTAAATGGGA
7	β-actin	CGGTGCTGAGTATGTCTGTG	GGTGGAGATGATGACCCTTT
